# Downregulation of Salusin-β protects renal tubular epithelial cells against high glucose-induced inflammation, oxidative stress, apoptosis and lipid accumulation via suppressing miR-155-5p

**DOI:** 10.1080/21655979.2021.1972900

**Published:** 2021-09-04

**Authors:** Hongmei Chen, Genjuan Jin

**Affiliations:** a Department of Endocrinology and Metabolism, Nantong No.2 People's Hospital, Nantong, Jiangsu Province, China; b Department of Endocrinology, Zhejiang Xiaoshan Hospital, Hangzhou, Zhejiang Province, China;

**Keywords:** Salusin-β, miR-155-5p, renal tubular epithelial cells, diabetic nephropathy

## Abstract

Diabetic nephropathy (DN) is the main contributor to the excess mortality for patients suffering from diabetes. Here, C57BL/6 mice received 4 weeks of high-fat diet and intraperitoneal injection of STZ (100 mg/kg). Mice with random blood glucose level ≥16.7 mmol/L and positive urine protein were recognized as successful DN model. To construct an *in vitro* model, HK-2 cells were incubated with 30 mM glucose. RT-qPCR and western blot were employed to measure Salusin-β levels in kidney tissues of DN mice and HG-induced HK-2 cells. Meanwhile, RT-qPCR was performed to detect miR-155-5p level in kidney tissues of DN mice and HG-induced HK-2 cells. TNF-α, IL-6, IL-1β, ROS, SOD and CAT levels were assessed using commercial assay kits. Furthermore, apoptosis of HK-2 cells was assessed via flow cytometric analysis and TUNEL staining. In addition, intracellular lipid accumulation and total cholesterol levels were detected using Oil red O staining and TC ELISA kit. Herein, Salusin-β and miR-155-5p levels were distinctly upregulated in kidney tissues of DN mice and HG-induced HK-2 cells. Downregulation of Salusin-β reduced miR-155-5p expression. Salusin-β silencing dramatically relieved inflammatory and oxidative injury, suppressed apoptosis as well as lipid accumulation induced by HG in HK-2 cells. Besides, miR-155-5p elevation partially abrogated the alleviating effects Salusin-β silencing on HG-induced RTEC injury. In summary, downregulation of Salusin-β protected HK-2 cells against HG-induced inflammation, oxidative stress, apoptosis and ameliorated lipid accumulation through suppressing miR-155-5p, which indicated that Salusin-β could be a potential therapeutic drug for DN.

## Introduction

Diabetes, the third most serious chronic disease worldwide after tumors and cardiovascular diseases, poses a threat to human health [1]. Accompanied by the change of modern people's lifestyle, the occurence rate of diabetes has been increasing year after year [2]. On the one hand, diabetic nephropathy (DN) is one of the most ordinary chronic complicating diseases of diabetes, on the other hand, it is also the principal contributor to the incidence of end-stage renal disease (ESRD) [3]. Besides, long-term hyperglycemia can cause renal metabolic disorders and hemodynamic changes, exacerbating the situation of DN [4]. Hence, it's of great significance to comprehend the pathogenesis and develop new diagnostic and therapeutic targets of DN.

Renal tubule epithelial cells play an indispensable part in glomerular filtration barrier [5]. The structural and functional integrity of renal tubule epithelial cells have close relation with the maintenance of glomerular filtration function [6]. Dysfunction of renal tubule epithelial cells would result in interstitial fibrosis, glomerulosclerosis as well as proteinuria. What’s worse, they may even damage renal function and accelerate the progression of DN [7]. In recent years, increasing data has confirmed that inflammation and oxidative stress are closely related with the incidence and advancement of DN [8,9]. Thus, the effective target to alleviate the damage of renal tubule epithelial cells must be sought.

MicroRNA (miRNA), as an ordinary small non-coding RNA, is widely involved in gene post-transcriptional as well as epigenetic regulations [10]. The abnormal miRNA expression has tremendous influence on the occurrence and advancement of DN, mediating the pathophysiological changes such as renal tissue fibrosis, inflammation, podocyte apoptosis in DN [11,12]. Additionally, it is confirmed that miR-155-5p gains an obvious increase in HG-injured HK-2 cells and the renal tubules of DN patients [13,14]. Researches above implies that miR-155-5p has a promising future to mechanically mediate the progression of DN.

Salusin-β, a new type of cardiovascular bioactive peptide, has the biological functions of relaxing blood vessels and promoting cell proliferation [15]. Salusin-β has been proved to enhance miR-155-5p expression and silenced miR-155-5p can impede the functions of Salusin-β on VSMCs [16]. Additionally, Salusin-β expression has been identified to enjoy a rapid growth in high-glucose/high-fat-incubated HUVECs [17].

In spite of above backgrounds, little is known about the biological function of Salusin-β on DN. This current work was designed to investigate the precise effects of Salusin-β on the pathological manifestations of DN and to explore the underlying molecular mechanism. Herein, we observed elevated levels of Salusin-β and miR-155-5p in the kidney tissues of DN mice and HG-induced renal tubular epithelial cell (RTEC). Moreover, a positive regulation between Salusin-β and miR-155-5p expression was confirmed. Findings concluded that Salusin-β silencing could alleviate HG-induced RTEC injury by suppressing miR-155-5p expression, highlighting the roles of Salusin-β and miR-155-5p in DN therapies.

## Materials and methods

### Preparation of diabetic animal model

The 8-week-old C57BL/6 male mice, obtained from Shanghai SLAC Laboratory Animal Co. Ltd, were placed under a temperature- and humidity-controlled environment (a 12-h/12-h light/dark cycle) with supply of water and standard chow. Establishment of diabetic animal model was based on 4 weeks of high-fat diet (HFD; 22% fat, 48% carbohydrate and 20% protein) feeding and intraperitoneal injection of streptozotocin (STZ; Solarbio, Beijing, China) solution with the dose of 100 mg/kg body weight. 72 h after the injection, the random blood glucose level ≥16.7 mmol/L were recognized as successful diabetic animal models [18]. Kidneys, blood and urine samples were harvested for subsequent analyses. All procedures performed in this study were approved by the Ethics Committee of Nantong No.2 People’s Hospital.

### Biochemical index detection

The blood/urine samples were collected to measure corresponding biochemical parameters. The serum creatinine (Scr), blood urea nitrogen (BUN), urinary albumin and urinary creatinine were detected by an automatic biochemical analyzer (Hitachi, Tokyo, Japan). Urine albumin creatinine ratio (ACR) = urinary albumin (μg)/urinary creatinine (mg).

### Cell culture

American Type Culture Collection (ATCC, VA, USA) was the provider of renal tubular epithelial cells (HK-2). These cells were fostered in RMPI-1640 medium (HyClone, UT, USA) with the mixture of 10% fetal bovine serum (FBS; Gibco, NY, USA), 100 U/ml penicillin and 100 µg/ml streptomycin (Invitrogen; CA, USA). Cells were kept at 37°C in a humidified environment containing 5% CO_2_.

### Cell treatment

HK-2 cells were fostered under following cases, including normal glucose (NG, 5.5 mM), mannitol (MA, 5.5 mM glucose+24.5 mM MA) or high glucose (HG, 30 mM). HK-2 cells incubated with 30 mM glucose (HG) were used as an *in vitro* model [19].

### Cell transfection

For transfection, sh-NC, miR-NC, sh-Salusin-β-1/2 as well as miR-155-5p mimic were provided by Shanghai GenePharma. The sequences of sh-Salusin-β and miR-155-5p mimic were as follows: sh-Salusin-β: 5'-gatccGCCCTTCTTGGGTTGTGTATGTTCAAGAGACATACACAACCCAAGAAGGGCTTTTTTa-3' (sense), and 5'-agcttAAAAAAGCCCTTCTTGGGTTGTGTATGTCTCTTGAACATACACAACCCAAGAAGGGCg-3' (antisense); scrambled shRNA: 5'-gatccGTTCTCCGAACGTGTCACGTTTCAAGAGAACGTGACACGTTCGGAGAACTTTTTTACGCGTg-3' (sense), 5'-aattcACGCGTAAAAAAGTTCTCCGAACGTGTCACGTTCTCTTGAAACGTGACACGTTCGGAGAACg-3' (antisense); miR-155-5p mimic: 5ʹ-UUAAUG CUAAUCGUGAUAGGGGUU-3ʹ; miR-NC: 5'-UCUACUCUUUCUAGGUUGUGG-3ʹ. The shRNAs (MOI = 100) and/or mimics (50 nM) were transfected into HK-2 cells by using Lipofectamine 2000 (Invitrogen, CA, USA) in line with the protocol put forward by manufacturer.

### Enzyme-linked immunosorbent assay (ELISA)

With application of corresponding ELISA kits (R&D Systems, MN, USA), the levels of inflammatory cytokines tumor necrosis factor-α (TNF-α), interleukin-1β (IL-1β) and interleukin-6 (IL-6) were tracked in accordance with the instructions recommended by manufacturer. The optical density (OD) value at 450 nm was read and calculated based on the standard curve.

### Reactive oxygen species (ROS) measurement

HK-2 cells were incubated with 40 µM 2', 7'-dichlorofluolescein diacetate (DCFH-DA) for 30 min at 37°C. After incubation, HK-2 cells were rinsed with serum-free medium. Then, the fluorescence intensity was measured at 488 nm excitation and 525 nm emission wavelength with the use of the fluorescence microplate reader (Bio-Rad, CA, USA).

### Measurement of superoxide dismutase (SOD) and catalase (CAT) activities

Centrifugation was adopted to gather the supernatant of HK-2 cells. In line with the protocol recommended by manufacturer, the activities of SOD (Solarbio, Beijing, China) and CAT (Solarbio, Beijing, China) were tracked using commercial kits.

### Measurement of total cholesterol

After washing with PBS, HK-2 cells were lysed with lysis buffer and then centrifuged at 2000 rpm for 10 min. On the basis of the instructions raised by manufacturer, the supernatant was applied to measure the intracellular total cholesterol level by a TG ELISA kit (Applygen, Beijing, China).

### Annexin V-FITC/propidium iodide (PI) staining

HK-2 cells were plated in six-well plates at the density of 1 × 10^6^ cells per well. After the designed treatment and transfection, cells were collected and washed twice with PBS. Then, cells were incubated with Annexin V-FITC and PI (Nanjing KeyGene Biotech Co., Ltd. Nanjing, China) for 15 min at room temperature in the dark. The apoptotic rate was determined by a flow cytometry (BD Biosciences, CA, USA). Cells were sorted into intact (Annexin V and PI double-negative), early apoptotic (Annexin V-positive), late apoptotic (Annexin V and PI double-positive) and necrotic (PI-positive) cells. Total apoptotic rate was calculated by combining early and late apoptotic cells.

### TUNEL staining

In short, HK-2 cells were cultured with 4% paraformaldehyde (PFA), permeabilized with 0.5% Triton X-100 and blocked with 5% bovine serum album (BSA). Then, cells were stained with TUNEL reagent for 60 min and counterstained with DAPI (1 μg/ml) for 10 min in the dark, followed by observation under a florescent microscope (Olympus, Tokyo, Japan). The apoptotic rate was determined by calculating the percentage of the number of positive apoptotic cells in the total number of nuclei.

### Oil red O staining

As the designed treatment and transfection completed, the collection of dish climbing glasses of cells were ready to conduct. HK-2 cells were fixed with 4% PFA, washed with 60% isopropanol and then stained with Oil red O for 30 min. After HK-2 cells were rinsed with PBS, a light microscope was applied to count the number of the positive ones.

### Real-time quantitative PCR analysis

Based on the manufacturer’s instructions, Trizol reagents (Invitrogen, NY, USA) was used for isolating the total RNA from cells or tissues. Equivalent amounts of RNA samples were used to synthesize cDNA with the application of cDNA Synthesis kit (Takara, Japan). Real-time PCR for gene quantitation assay using SYBR-Green Supermix (Invitrogen, NY, USA) was performed on the ABI PRISM 7000 Sequence Detection System (ABI/Perkin Elmer, Foster City, CA). The primer sequences for PCR assay: Salusin-β forward, 5'-TCACTTCTCTCCTATCATCCACTCC −3' and reverse, 5'- GGCAGCTTGTCCATCTCATCG −3'; miR-155-5p forward, 5'- ACACTCCAGCTGGGTTAATGCTAATCGTGATA −3' and reverse, 5'- CTCAACTGGTGTCGTGGA −3'; U6 forward, 5'- CTCGCTTCGGCAGCACA −3' and reverse, 5'- AACGCTTCACGAATTTGCGT −3'; GAPDH forward, 5'- GTGGAGTCTACTGGCGTCTT −3' and reverse, 5'- TGCTGACAATCTTGAGGGA −3'. PCR reaction conditions were as follows: 95°C for 10 min, followed by 40 cycles of 95°C for 15 sec and 65°C for 60 sec. The mRNA levels were normalized to GAPDH or U6 using the 2^−ΔΔCq^ method [20].

### Western blotting analysis

RIPA lysis buffer (Beyotime, Shanghai, China), which contains protease inhibitors, was employed to isolated the total protein from the cultured cells or tissues. In line with the instructions required by manufacturer, BCA Protein Assay Reagent Kit was adopted to detect the protein concentration of the supernatant. Subsequently, equal amounts of protein samples were separated by sodium dodecyl sulfate-polyacrylamide gels. After separation, the proteins were transferred onto PVDF membranes (Millipore Corporation, Billerica, MA, USA) and blocked with 5% skimmed milk in PBS. Salusin-β (1:200, B-010-68, Phoenix Pharmaceuticals Inc.) and GAPDH (1:5000, ab8245, Abcam), the appropriate primary antibodies, were used for incubation of the membranes. An enhanced chemiluminescence (ECL) was put into use with the aim of visualizing the immunoreactive bands, meanwhile, the density of protein bands was assayed with the application of Image J software.

### Statistical analysis

Data from three independent experiments were presented as mean ± standard deviation (SD). SPSS software (Version 16.0; SPSS, Chicago, IL, USA) was employed to perform data analysis. Differences among diverse groups were evaluated by one-way ANOVA analysis followed by Tukey’s Multiple Comparison post-hoc test. Value of p < 0.05 was considered to be statistically significant. *P < 0.05, **P < 0.01, ***P < 0.001.

## Results

### High levels of Salusin-β and miR-155-5p in the kidney tissues of DN mice and HG-induced renal tubular epithelial cells

**Figure 1.**
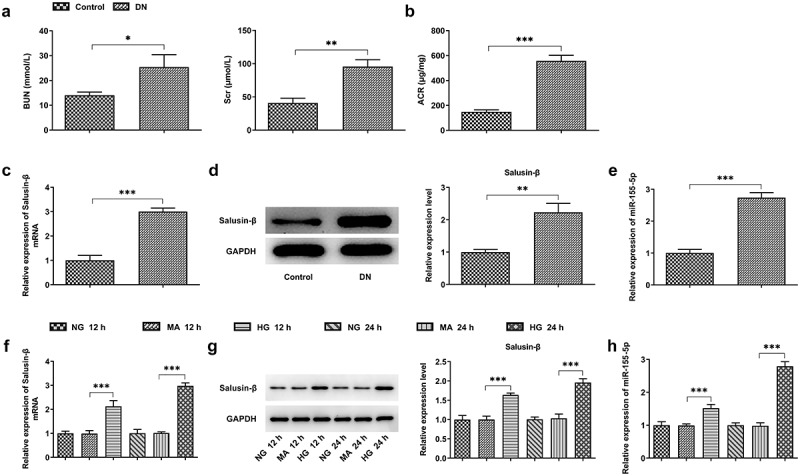
High levels of Salusin-β and miR-155-5p in the kidney tissues of DN mice and HG-induced renal tubular epithelial cells (HK-2 cells). (a) Levels of BUN and Scr in serum samples. (b) Level of ACR in urine samples. (c) RT-qPCR analysis of Salusin-β mRNA level in the kidney tissues of mice. (d) Western blotting analysis of Salusin-β protein level in the kidney tissues of mice. (e) RT-qPCR analysis of miR-155-5p level in the kidney tissues of mice. (f) RT-qPCR analysis of Salusin-β mRNA level in HK-2 cells. (g) Western blotting analysis of Salusin-β protein level in HK-2 cells. (h) RT-qPCR analysis of miR-155-5p level in HK-2 cells

Establishment of the diabetes mellitus model was built on the basis of high-fat diet for 4 weeks and intraperitoneal injection of streptozotocin solution with the dose of 100 mg/kg body weight. Mice with random blood glucose ≥16.7 mmol/L were recognized as successful diabetic animal models. Additionally, the blood and urine samples were harvested for analyses of biochemical index. Higher levels of BUN, Scr and ACR were observed in mice assigned to DN group, indicating the successful establishment of DN model (Figure 1(a, b)). Results of RT-qPCR and western blotting analysis strongly proved that Salusin-β expression gained significant enhancement in the kidney tissues of DN mice (Figure 1(c, d)). At the same time, in comparison with that in control, miR-155-5p level was tremendously enhanced in the kidney tissues of DN mice (Figure 1(e)). These results above suggested that Salusin-β and miR-155-5p may be involved in DN pathology. Then, to construct an *in vitro* model, 30 mM glucose (HG) was used to incubate quiescent HK-2 cells for 12 or 24 h. The mannitol (24.5 mM) served as an osmotic control. In a time-dependent manner, treatment with 30 mM glucose (HG) dramatically increased Salusin-β protein and mRNA levels (Figure 1(f, g)) and miR-155-5p level (Figure 1(h)).

### Downregulation of Salusin-β reduced miR-155-5p expression

**Figure 2.**
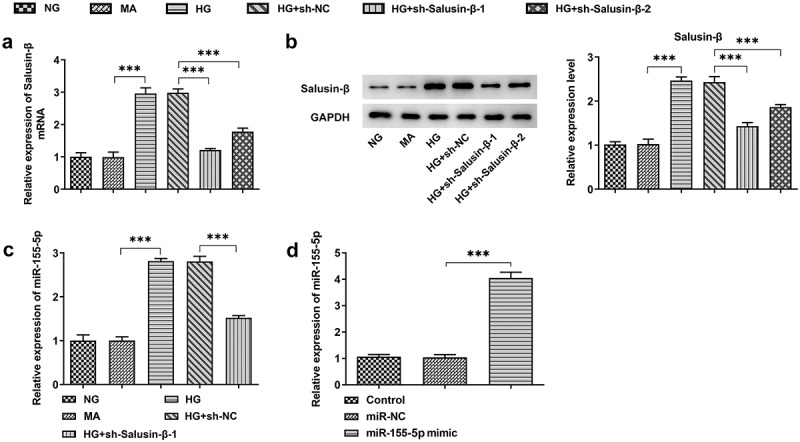
Downregulation of Salusin-β reduced miR-155-5p expression. (a) RT-qPCR analysis of Salusin-β mRNA level in HG-induced HK-2 cells that were transfected with sh-Salusin-β-1 or sh-Salusin-β-2. (b) Western blotting analysis of Salusin-β protein level in HG-induced HK-2 cells that were transfected with sh-Salusin-β-1 or sh-Salusin-β-2. (c) RT-qPCR analysis of miR-155-5p level in HG-induced HK-2 cells that were transfected with sh-Salusin-β-1. (d) RT-qPCR analysis of miR-155-5p level following transfection with miR-155-5p mimic

Based on time-dependent expression differences of Salusin-β in HG-induced HK-2 cells, HG treatment for 24 h was selected for the following studies. Transfection efficiency was examined by performing RT-qPCR and western blotting analysis, and the results indicated that Salusin-β levels in HG-induced HK-2 cells were markedly declined by transfection with sh-Salusin-β-1 or sh-Salusin-β-2 (Figure 2(a, b)). Then, sh-Salusin-β-1 was chosen for the subsequent experiments due to its optimal efficiency. Moreover, miR-155-5p level enhanced by HG was obviously suppressed following sh-Salusin-β-1 treatment (Figure 2(c)). In addition, transfection of miR-155-5p mimic into HK-2 cells was operated with the aim of elevating miR-155-5p level for the subsequent experiments. Meanwhile, RT-qPCR analysis was conducted to check the transfection efficiency (Figure 2(d)).

### Downregulation of Salusin-β alleviated HG-induced inflammatory injury and oxidative stress by suppressing miR-155-5p

**Figure 3.**
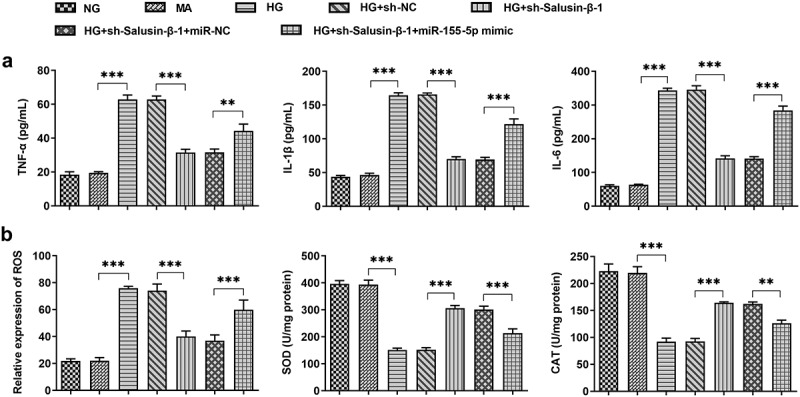
Downregulation of Salusin-β alleviated HG-induced inflammatory injury and oxidative stress by suppressing miR-155-5p. (a) The release of inflammatory cytokines including TNF-α, IL-1β and IL-6 were measured using ELISA Kits. (b) Measurement of ROS, SOD and CAT activities

In HG-induced HK-2 cells, inflammatory injury and oxidative stress could be observed clearly. The decreased release of inflammatory cytokines including IL-1β, IL-6 as well as TNF-α demonstrated that downregulation of Salusin-β could relieve HG-induced inflammatory injury. Besides, overexpression of miR-155-5p induced the release of inflammatory cytokines, at the same time, it eliminated the suppressive effects of Salusin-β silencing on inflammation (Figure 3(a)). Additionally, after co-transfection of miR-155-5p mimic, the level of ROS gained a distinct increase while the levels of SOD and CAT were decreased, suggesting that miR-155-5p overexpression exacerbated oxidative stress that was inhibited by Salusin-β silencing (Figure 3(b)).

### Down-regulation of Salusin-β repressed the apoptosis of HG-induced HK-2 cells by suppressing miR-155-5p

**Figure 4.**
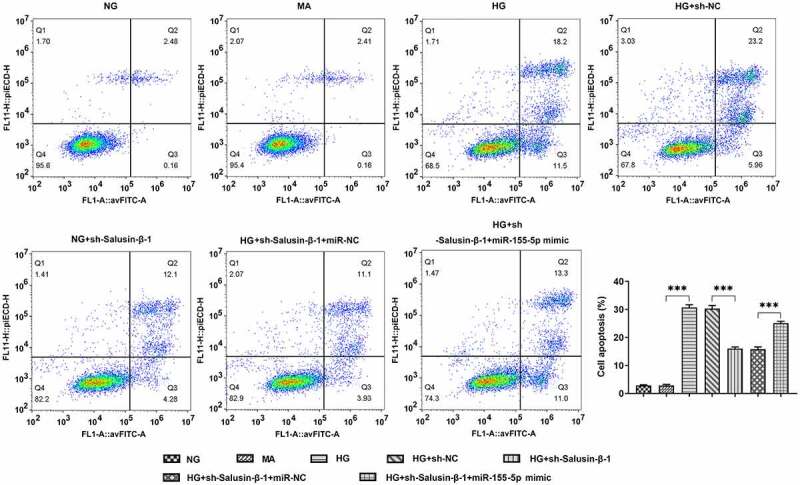
Downregulation of Salusin-β repressed the apoptosis of HG-induced HK-2 cells by suppressing miR-155-5p. Flow-cytometric analysis for detection of the apoptotic rate of HK-2 cells

**Figure 5.**
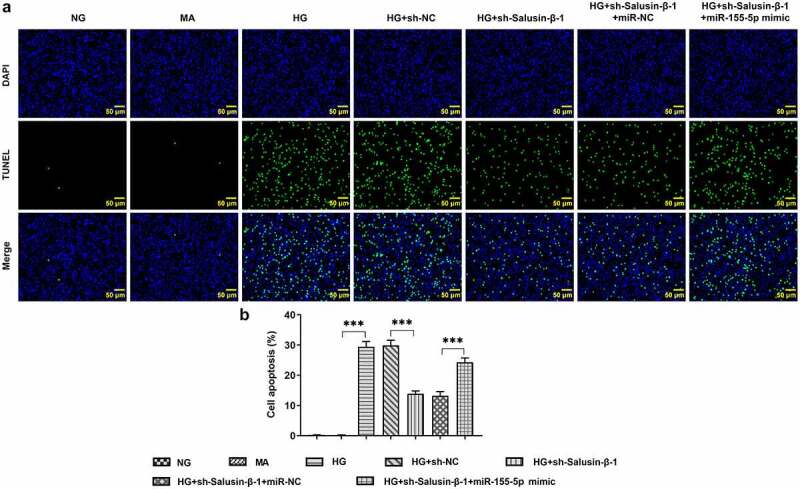
Downregulation of Salusin-β repressed the apoptosis of HG-induced HK-2 cells by suppressing miR-155-5p. (a) TUNEL staining for the assessment of cell apoptosis. (b) Quantitative analysis of cell apoptosis

HG-induced HK-2 cells received designed transfection, the apoptotic rate of which was assessed with the application of flow cytometric analysis and TUNEL staining. Cell apoptosis was dramatically aggravated upon HG treatment. Results of flow cytometric analysis discovered that Salusin-β silencing significantly reduced cell apoptosis, which was partially reversed by upregulation of miR-155-5p (Figure 4). Moreover, the distinct elevation of TUNEL positive cells following co-transfection with miR-155-5p mimic clearly evidenced that miR-155-5p overexpression could aggravate cell apoptosis suppressed by Salusin-β silencing (Figure 5(a, b).

### Downregulation of Salusin-β impeded lipid accumulation in HG-induced HK-2 cells by suppressing miR-155-5p

**Figure 6.**
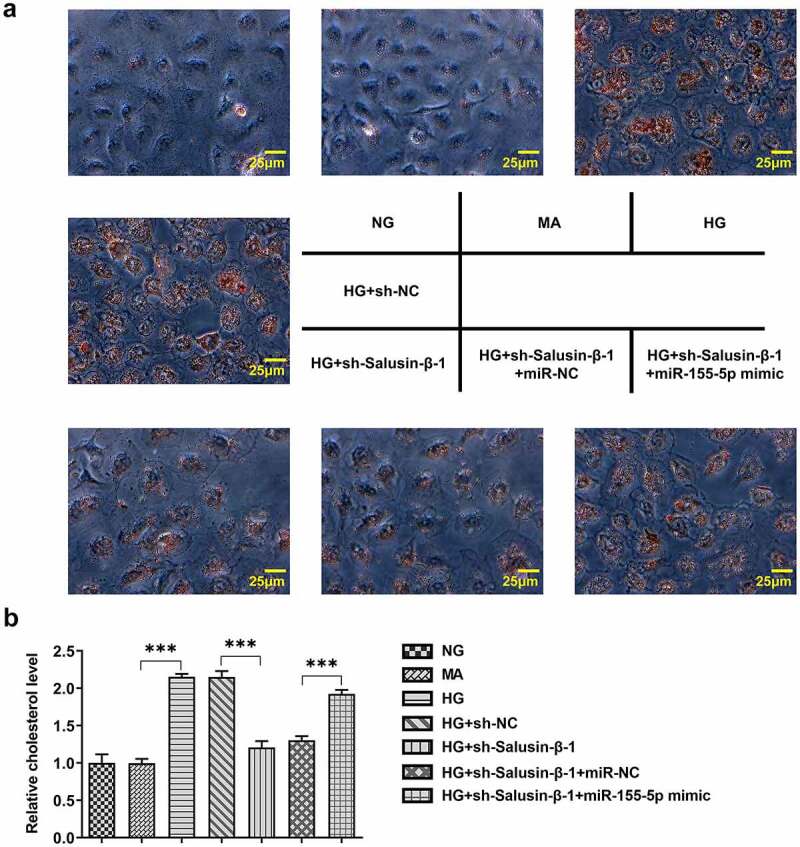
Downregulation of Salusin-β impeded lipid accumulation in HG-induced HK-2 cells by suppressing miR-155-5p. (a) Oil red O staining of the intracellular lipid droplet. (b) Relative TG concentrations

In order to investigate the role of Salusin-β in lipid metabolism, Oil red O staining and TG concentration measurement were adopted so as to measure the intracellular lipid contents. Lipid droplets and cholesterol levels rapidly increased in HK-2 cells under HG stimulation. Compared with HG treatment, less lipid droplet formation and lower cholesterol level were observed following transfection with sh-Salusin-β-1. Besides, reintroduction of miR-155-5p mimic partially reversed this inhibitory effect of sh-Salusin-β-1 on lipid accumulation (Figure 6(a, b).

## Discussion

DN is recognized as one of the most serious chronic complications featuring progressive proteinuria and renal failure, eventually developing into ESRD [3]. In recent years, the prevalence of DN has dramatically increased worldwide. Moreover, it has become a serious public health problem threatening human health [21]. What’s worse, the poor therapies for DN results in high possibility of mortality at home and broad [22]. It has been testified that the existing therapeutic drugs can protect the renal function of DN to a certain degree while fail to delay or control the pathogenesis of DN [23]. Therefore, the exploration of accurate and feasible drugs or therapeutic targets is the focus of current medical research.

MicroRNA belongs to a kind of non-coding single-stranded small-molecule RNA [10]. Recently, it has been confirmed that miRNA is of great value in the early diagnosis and prognosis judgment of DN [11]. For instance, Kato et al. reported that miR-192 was highly expressed in the kidney tissues of DN and played an important role in the development of DN [24]. Wang et al. revealed that as the glucose concentration in HK-2 cells increased, miR-155-5p was also increased accordingly [13]. In addition, miR-155-5p was proved to be upregulated in the renal tubules of DN patients [14]. In this research, miR-155-5p was discovered to gain tremendous upregulation in the kidney tissues of DN mice and HG-induced RTEC, which revealed the potential involvement of miR-155-5p in DN progression.

Salusin-β has been confirmed to be widely distributed in kidney and increased in diabetic aortic tissues and high-glucose/high-fat- incubated HUVECs [17,25]. In like wise, results of this research illustrated that Salusin-β expression in the kidney tissues of DN mice and HG-induced RTEC gained obvious upregulation. Sun et al indicated that the level of miR-155-5p could be enhanced by Salusin-β [16]. Similarly, we also discovered that downregulation of Salusin-β could inhibit miR-155-5p expression in RTEC, demonstrating a positive regulation between Salusin-β and miR-155-5p.

RTEC is the major cell type in renal tubulointerstitium. On the one hand, RTEC injury directly results in acute renal failure, on the other hand, it is also the main contributor to the renal interstitial fibrosis (RIF) and the common pathological process of irreversible ESRD [26]. The prevention and treatment of RTEC injury plays an indispensable role in slowing down and reversing the progress of RIF. In this research, the findings concluded that Salusin-β deficiency repressed inflammation, oxidative stress, apoptosis and lipid accumulation in HG-treated RTEC. However, miR-155-5p elevation boosted RTEC injury, partially reversing the influence of Salusin-β silencing on inflammation, oxidative stress, apoptosis and lipid accumulation in HG-stimulated RTEC. Mechanically, downregulation of Salusin-β alleviated HG-induced RTEC injury by suppressing miR-155-5p expression.

## Conclusion

To sum up, our results revealed that Salusin-β might contribute to HG-triggered RTEC injury through promoting miR-155-5p expression. These findings may identify novel therapeutic drug or new biomarkers for DN therapy.

## Data Availability

The analyzed data sets generated during the present research are available from the corresponding author on reasonable request.
